# Ethical aspects of digital health from a justice point of view

**DOI:** 10.1093/eurpub/ckz167

**Published:** 2019-11-18

**Authors:** Caroline Brall, Peter Schröder-Bäck, Els Maeckelberghe

**Affiliations:** 1 Department of Health and Technology, Health Ethics and Policy Lab, ETH Zurich, Zurich, Switzerland; 2 Department of International Health, Care and Public Health Research Institute (CAPHRI), Maastricht University, Maastricht, The Netherlands; 3 Section Ethics in Public Health, European Public Health Association (EUPHA); 4 Institute for Medical Education, University of Groningen, University Medical Center Groningen, Groningen, The Netherlands

## Abstract

Digital health is transforming healthcare systems worldwide. It promises benefits for population health but might also lead to health inequities. From an ethical perspective, it is hence much needed to adopt a fair approach. This article aims at outlining chances and challenges from an ethical perspective, focusing especially on the dimension of justice—a value, which has been described as the core value for public health. Analysed through the lenses of a standard approach for health justice—Norman Daniels’ account of just health and accountability for reasonableness—most recent and relevant literature was reviewed and challenges from a justice point of view were identified. Among them are challenges with regard to digital illiteracy, resulting inequities in access to healthcare, truthful information sharing to end users demanding fully informed consent, dignity and fairness in storage, access, sharing and ownership of data. All stakeholders involved bear responsibilities to shape digital health in an ethical and fair way. When all stakeholders, especially digital health providers and regulators, ensure that digital health interventions are designed and set up in an ethical and fair way and foster health equity for all population groups, there is a chance for this transformation resulting in a fair approach to digital health.

## Introduction

Digital technology is already part of our daily lives. We use smartphones to navigate our routes and order our purchases. Also in the field of health, the digital dimension is ever increasing, and in the last few years, digital health initiatives received much interest and increasing investments from public and private sources.

The purposes and utilizations of digital health are to monitor, prevent, screen, diagnose and treat health-related issues on the healthcare and public health level. Digital health methods are increasingly embraced to strengthen health systems worldwide, as for instance put forward in the recently published recommendations on digital interventions for health system strengthening.^1^ Kickbusch terms this ongoing digital transformation within health and medical care as ‘health 4.0’,^2^ highlighting the importance of adjusting existent practice and governance structures to meet the challenges implicated by digital health, as for instance how data should be stored and accessed by whom, who can benefit from digital health and who is at risk of being excluded, and which types of informed consent should be employed. In view of this change of cultural environment, it is important to carefully consider the chances and challenges from an ethical perspective in order to establish and frame a sound and fair approach for digital health. Yet, publications that sketch the ethics of digital health are still scarce, given this is an innovative field of public health. Thus, in the following we will outline these chances and challenges from an ethical perspective, focusing especially on the dimension of justice—a value, which has been described as the core value for public health.^3^ Justice is closely linked to and addresses ‘questions of responsibilities and obligations’^4^ when it comes to balancing benefits and risks of population health interventions. More concretely, we define justice in line with Norman Daniels’ account of just health and accountability for reasonableness,^5^^,^^6^ which has been considered the ‘most well-known rationale’^7^ for health and fairness. The argument he started developing more than 30 years ago has been considered a ‘seminal’ and ‘classic’^8^ work. It is considered a standard approach and among the ‘key narratives (and vocabulary)’^9^ in public health ethics, in research and teaching.

Daniels argues that justice describes the social obligations to promote and restore health as a means to achieve individual opportunities and exercise individual autonomy.^10^ He specifies that everyone should have fair access to public health and healthcare to have fair equality of opportunities in society, resulting in health equity.^5^ Daniels also states that fair processes are needed to ensure legitimacy and fairness. His concept of accountability for reasonableness declares that policies should be made in a transparent way, based on reasonable arguments and with the option of being revised.^6^ Such a public health justice approach towards the implications—the chances and challenges—of digital health can uncover what is ethically at stake, where responsibilities lie for those involved, and can guide and justify resulting policy choices.

Thus, with this understanding of a public health justice approach, we discuss the ethical chances and challenges unfolding in digital health. We base our analytic overview of these issues on a narrative review in order to obtain a broad perspective on recent and relevant literature on digital (public) health. We point out what ethical guidance is needed and for whom, and finally we address existing policy and practice initiatives to foster ethical digital health.

## Ethical chances and challenges of digital health

The sphere in which ethical issues in digital health proliferate is multidimensional. First, it is dependent on the distinct phases of digital health usage, i.e. before accessing digital health technologies, during as well as after usage. Second, different stakeholders from the medical and non-medical, public and private arena are involved, setting new challenges with regard to governance structures, emphasizing the need for rethinking responsibilities. Third, challenges are on the one hand tied to technical issues, such as how to protect data (e.g. secure storage, firewalls, etc.). On the other hand, they are tied to aspects related to general governance (as for instance accountability and transparency). Besides these challenges, which can even result in physical, psychological or social harms to individuals,^11^ there are also chances for using digital health to establish fairer health systems. We will address these challenges and chances (mentioned in the literature), following their occurrence during the distinct phases of digital health usage.

### Before utilization of digital health

#### Access

The first phase of digital health usage is before users actually access such technologies and applications, where ethical considerations inherently arise in line with aspects related to access. They specifically centre around logistic and resource-related aspects, including equitable access to digital health services in terms of affordability of and access to technological equipment.^12^ Here also the availability of such services plays a role: for underserved communities and populations, for instance people suffering from rare diseases, elderly or homeless, digital health services might not be offered or even developed. It remains crucial to safeguard fairness and equity in access already when developing such digital health approaches.^13^ Integrating such ethical considerations in the planning phase is—mostly in the field of artificial intelligence for health—referred to as ‘ethics by design’.^14^ The developers of digital health interventions hence have a moral responsibility to design such technologies in a way that take into account ethical forethoughts and aspects, for instance when designing algorithms for artificial intelligence, that represent all parts of the population and leave no ground for bias and resulting discrimination.

In general, the employment of digital health technologies can give rise to inequalities in access which go beyond affordability of technology, but depend on the individual’s technological ability and capacity to engage with e-health tools. When certain populations are excluded to use such technologies, for instance due to age-related socialization and sometimes corresponding digital illiteracy, the danger of an unjust *health system 4.0* is prevalent. However, digital health technologies also offer chances for inclusion of population groups which experience barriers to access conventional healthcare provision, for instance due to geographical distance to reach medical settings in general or specified healthcare professionals, or due to physical inability to travel to the medical sites on a regular basis. Here, digital health can be seen as an enabler for fair and accessible health provision by extending healthcare coverage to areas and persons with previously limited access to health services or research.^15^ This, again, can save overall healthcare costs through efficiency improvements and provides a more demand-oriented provision of healthcare services. Also, possible increases in coverage contribute to improve global health and can be evaluated as a measure to improve equality of opportunity.

#### Truthful information, empowerment and informed consent

In order to make people capable to actually use the opportunities offered to them if they wish, truthful information about the benefits and risks of engaging in digital health methods has to be provided to the individual users. Hence, users should be motivated and empowered (in an informational as well as technical sense) to engage in digital health technology. For this, open communication, technical training and education should be offered. It is important that their participation is voluntary and is not undermined by any sort of incentive, be it of financial nature or prioritizing those that use digital health technologies when they seek medical care in non-digital, conventional healthcare settings. And not using these opportunities may not be sanctioned or result in a lack of access to health services. Moreover, ‘users’ should be aware that their data are being collected for health-related purposes, for instance in the case of location tackers, which can give information about an individual’s health (e.g. when frequent visits to hospitals or other healthcare sites are documented). Yet, for public health purposes, aggregate information, e.g. from social media posts about flu symptoms, could give hints of the spread of diseases—techniques being referred to as digital epidemiology and epidemic forecasting. In general, however, there is the danger of digital health establishing a surveillance society. This and other contested uses should be prohibited by law and prevented in practice.

As regards truthful information, informed consent also plays a major role. Whereas traditional models of informed consent aimed to inform patients and research subjects and primarily focused to avoid harm to the individual in the course of the procedure—thus having a limited time span—new models of informed consent for digital health have to be considered. Those new models should not only take into account intended and unintended uses of data provided by aware users, but should also consider the larger time dimension, when data are stored (and potentially used) for a considerable amount of time. Additionally, certain types of digital health, e.g. when genetic data are involved, extend the knowledge gained about an individual to his or her genetically related family members. Revision of existing and traditional models of informed consent, such as opt-out, waiver, no consent and open or categorical consent, is needed for meeting the challenges posed and adjusting consent mechanisms accordingly to ensure and promote autonomy for everyone in line with fair data uses.^16^

### During utilization of digital health

#### Fairness in storage, access, sharing and ownership

During the phase of actual utilization of digital health technologies and also thereafter, challenges of ethical concern arise with regard to storage, access, sharing and ownership of data as well as return of results. Apart from touching relevant ethical considerations in line with security, privacy, confidentiality, discrimination, unintended uses of data and right to know or not to know results about sometimes incidental findings, these aspects also have implications for a fair use of digital health.

Initially, data have to be stored in such a way that no unauthorized access through hacking or other fraud is facilitated that allows for discrimination and stigmatization, when confidential information is falling into the wrong hands. Also, when data collectors grant access to other stakeholders, various considerations with view to fairness emerge: what is the purpose of accessing and using the data? What is the benefit for providing and accessing stakeholders? Do they pursue commercial objectives or benefit for the public? Are users aware of the uses of their data? And are these data only used for intended purposes or also unintended uses? These questions are relevant to address as they not only touch the ethical issues of autonomy, informed choice and right to privacy, but are also closely interlinked with justifiable uses of data basing on the individual’s right to determine for what his or her personal information is used for.

A fair use of data should furthermore be guaranteed as regards ownership of data, circling around questions to be answered as regards to who owns the data and who is custodian of data: data collectors, users themselves, governments, public organizations, etc. Although no universal regulation has been established yet,^17^ it should be guaranteed that individuals who donate their data are not exploited. It also remains to be regulated who should be eligible to financially benefit from donated data under what conditions (and to what extent). Despite the financial benefit, there is nevertheless a benefit in terms of welfare for the public. According to Topol a ‘democratization of medicine’ is supported by digital health, granting individuals increased access to their medical information, which increases their freedom to direct their health more autonomously.^18^ What can be an advantage for some, also has to be regarded with caution so that those who are rather unable to manage their own health are not overburdened.

#### Dignity and autonomy

Moreover, digital health tools should only be applied when the dignity of the patient can be preserved. For instance in the case of using telemedicine in hospital settings, the conveyance of potentially bad news to the patient should be in accordance to upholding dignity of the patient and therefore distant technologies (through using screens) should be refrained from when delivering news which put the patient in a vulnerable situation. Instead, personal and face-to-face communication is preferred to protect dignity of patients in vulnerable situations. Here, however, autonomy—in terms of patients’ choice of the communication channel—can tailor the delivery of healthcare to patients’ needs. Conversely, patients who do not want to be institutionalized can stay at home longer and be better supported in their home environment by means of telemedicine. Their quality of life and dignity can thus be increased through the use of such technologies.

Although no all-encompassing account of the ethical issues surrounding digital health can be provided given that the field is still evolving and other questions of moral concern will be emerging, we set out issues which are pressing from a justice point of view. These concrete issues mentioned are reflected by ethical values (adapted from Royakkers et al. and extended by specifications of the Daniels framework of justice),^19^ which are based on Daniels’ account of justice and the discussion above. An overview of the ethical values involved in digital health and their exemplification of issues touched is provided in table 1.

**Table 1:** Overview of ethical values of digital health and exemplification of issues involved (adapted from Royakkers et al., 2018 and adjusted based on Daniels, 2008 and discussion above)^5^^,^^19^

Ethical values	Exemplification of issues involved
Justice	Equity in access, exclusion, equal treatment, non-discrimination, non-stigmatization, data ownership, empowerment
Autonomy	Freedom of choice, informed consent, awareness of data collection and use, right to (not) know results
Privacy	Data protection, confidentiality, data sharing, intended/unintended uses of data
Security	Data storage, safety of information, protection against unauthorized access and use of data
Responsibilities	Trust, balance of power, relation between stakeholders (e.g. user–government–provider), benefits and benefit sharing, data ownership
Procedural values	Transparency, accountability, inclusiveness

## What ethical guidance is needed and for whom?

In view of the array of ethical challenges arising in the application of digital health, guidance should be given. In order to clarify for whom guidance would be necessary, we first determine who are the stakeholders involved. Digital health is integrated in a complex network of different parties, involving not only the users and providers of digital health technologies and applications. While the range of providers can already vary widely, stemming from public or governmental sources to private companies such as app technology start-ups or pharmaceutical and medical device companies, other stakeholders comprise doctors, who are responsible for providing medical programme planning and realization, and researchers for data analytics. Further stakeholders are insurers, government entities, non-governmental organizations and society in general, who are usually the implementation partners for digital health interventions. Here, it remains crucial, that exploitation of end user data is prevented and data are only used for purposes, of which end users are aware of when consenting to their data collection. Surveillance of data by governments and screening data by insurance companies to reject people’s applications have to be avoided at all costs. Underlying values and current as well as expected governance mechanisms need to be systematically addressed in order to increase the adoption by end users or patients.

Herein, we deem two values to be key for establishing digital health interventions in line with Daniels’ account of justice on a broader scale: trust and empowerment. In previous digital health initiatives, as for example in the 100,000 Genomes Project or initiatives by the Academy of Medical Sciences,^20^ it was shown that building and maintaining trust among multiple communities was experienced as a main challenge. Focusing on raising awareness among and engaging the public, as well as building trust through open communication, a common language, ongoing conversation and partnership are considered important. By such open communication and partnerships, users are subsequently empowered. Another mechanism to empower users is to foster digital literacy. Digital literacy refers to the ‘capabilities and understanding required to allow an individual to effectively engage with a data-driven technology or the processes that surround its use’.^20^ When users are empowered and capable to use digital health technologies accurately, it can be regarded as a chance to realize their individual opportunities to health in line with Daniels’ account of justice.^5^

In general, all stakeholders resume responsibilities, rights and duties which are building an interdependent network. Especially those stakeholders who develop and provide digital health interventions inherit a special role with regard to ensuring a just use and implementation of digital health technologies. Attending to those responsibilities also increases their trustworthiness. Furthermore, trustworthiness can be increased by procedural values guiding governance for service providers and can emphasize what to focus on for fair digital health provision. The most important procedural values for enabling fair digital health are transparency, accountability and inclusiveness. Transparency about structures, underlying algorithms for digital health tools and stakeholders involved and their interests empowers users to make better-informed decisions about engaging with such interventions. Accountability structures and mechanisms can ensure that responsibilities can be traced and held accountable for.^21^ Last, but maybe most importantly, inclusiveness should be a key element in setting up digital health interventions, so that inequities are diminished.

## Policy and practice initiatives to foster *ethical* digital health

In view of the magnitude of ethical issues emerging with the application of digital health technology, policy initiatives are needed, which specifically address those concerns. Recently the ethical dimension gained increasing attention in the policy field as moral questions of artificial intelligence and underlying algorithms were publicly discussed and the need for regulation was expressed. At the EU level, this was met when the ‘Ethics guidelines for trustworthy AI’ were published in April 2019 by the independent high-level expert group set up by the European Commission. It puts forward seven premises or values to be met by AI technologies to be trustworthy, which are (i) human agency and oversight, (ii) technical robustness and safety, (iii) privacy and data governance, (iv) transparency, (v) diversity, non-discrimination and fairness, (vi) societal and environmental well-being and (vii) accountability.^22^

With regard to digital health specifically, the WHO concurrently released their above-mentioned ‘Recommendations on digital interventions for health system strengthening’, which assess the benefits, harms, acceptability, feasibility, resource use and equity considerations of digital health interventions.^1^ Whereas the WHO website pronounces that ‘digital health interventions are not a substitute for functioning health systems, and that there are significant limitations to what digital health is able to address’,^23^ such view is not balanced enough and slightly too pessimistic in our view. Instead, we support that digital health interventions and technologies should rather be seen as a useful addition to non-digital healthcare provision.

In order to support practice, WHO also implemented the Digital Health Atlas—an online platform to collect, monitor and coordinate digital health initiatives worldwide—and announced to establish a section on digital health ‘to enhance WHO’s role in assessing digital technologies and support Member States in prioritizing, integrating and regulating them’.^23^

Also, in the related field of regulating health data—often referred to as big data—criteria and proposals were developed: in 2016, the OECD Recommendation on Health Data Governance set out the need to establish a health data governance framework to encourage the use of personal health data to serve health-related public interest, privacy and security.^24^ What these developments in policy have in common, is that they reflect the need for guidelines and regulations for dealing with digital health technologies. Whereas the EU General Data Protection Regulation can be seen as a first binding legal step toward protecting data privacy,^25^ other questions are still unaddressed, for instance clear and universal regulations on who has ownership of collected data. Establishing regulations to manage the handling of digital health technologies and big data not only fosters users’ trust in digital health and thus adoption of it, but it can also contribute to a fair application and use of digital health. As long as digital health can be offered in a fair manner, its opportunities can exceed the challenges.

An initiative from outside the European Region, which could hold lessons for future action in Europe is the Montréal Declaration for a Responsible Development of Artificial Intelligence that has been launched in 2017.^26^ Its strength is that the declaration (which was published one year later in 2018) was developed by means of a public deliberation process, which involved over 500 citizens, experts and stakeholders from diverse backgrounds. Such initiative that involves citizens offers transparent policy-making that is also in line with Daniels’ approach to justice and accountability for reasonableness and should inform future European initiatives to foster ethical digital health.

In line with the aim of this article, this general discussion highlighted ethical chances and challenges from a justice point of view, which need to be taken more into consideration than is currently the case to ultimately design and implement fair policies. Justice and related ethical concepts within digital health are underdiscussed in the academic literature so far and overlooked in practice. Especially from a public health point of view where the anticipated and real impacts of these innovative technologies on population health and health equity are investigated, ethical analysis to further identify and remedy violations of justice, respect for autonomy and other key values outlined above needs to be adopted as a necessary and integral aspect of all research.

## Conclusion

Digital health technologies offer opportunities to reshape health systems by broadening health coverage and spreading health information and literacy. Moreover, healthcare costs can potentially be reduced and efficiency can be enhanced. Yet, digital health technologies also catalyze challenges with regard to digital illiteracy, resulting inequities in access and informed consent, which need to be met. Hence, it is crucial for all stakeholders, especially digital health providers, to ensure that the digital health interventions are designed and set up in an ethical and fair way, thus fostering equity in access and fair equality of opportunity for all population groups and taking into account the needs of disadvantaged groups. If the awareness of these ethical challenges is existent and designers of digital health are held accountable to act according to these considerations when designing and implementing digital health technology, digital health can be an opportunity for everyone. Digital health should improve the fair and just access to health prevention and care; and if this is guaranteed, digital health has the opportunity of ‘only’ improving healthcare and public health, as other innovations in the past as well. Then it should be regarded as ‘just digital health’.

## Funding

No external funding has been received.

*Conflicts of interest*: None declared.

**Key points**

- Fair and equitable access to digital health technologies and interventions offers chances to healthcare coverage, spread of health information and literacy, and potentially efficiency of care.
- The diversity and range of stakeholders in digital health calls for a clear demarcation of each stakeholders’ specific responsibilities in assuring an ethical and fair digital health.
- Regulations and policies focusing on ethical guidance are needed to foster fair, equitable and trustworthy digital health aiming to empower users.
